# Endothelial Nitric Oxide Synthase Gene Polymorphisms and the Risk of Hypertension in an Indian Population

**DOI:** 10.1155/2014/793040

**Published:** 2014-08-06

**Authors:** Priyanka Shankarishan, Prasanta Kumar Borah, Giasuddin Ahmed, Jagadish Mahanta

**Affiliations:** ^1^Regional Medical Research Centre, NE Region, ICMR, P.O. Box 105, Dibrugarh, Assam 786001, India; ^2^Department of Biotechnology, Gauhati University, Guwahati, Assam 781014, India

## Abstract

Genetic variants of eNOS gene play a significant role in the pathogenesis of hypertension. Many environmental factors have, also, been implicated in the aetiology of hypertension. We carried out an age-matched case-control study among adults. Hypertension was defined according to JNC-VII criteria and eNOS gene polymorphisms were determined by PCR and PCR followed by PCR-RFLP. eNOS intron 4 aa genotype (adjusted OR 6.81; 95% CI 2.29–20.25) and eNOS 894TT genotype (adjusted OR 7.84; 95% CI 2.57–23.96) were associated with the risk of hypertension. Tobacco users (either smoking/chewing or both) with eNOS intron 4 aa genotype (OR 14.00: 95% CI 1.20–163.37), eNOS 894GG genotype (OR 5.56: 95% CI 3.72–8.31), and eNOS T-786C CC genotype (OR 9.00: 95% CI 1.14–71.04) were at an increased risk of hypertension. Similarly a significant gene-environment interaction was observed between individuals consuming alcohol with eNOS intron 4 aa genotype (OR 12.00: 95% CI 1.20–143.73) and eNOS 894GG genotype (OR 1.95: 95% CI 1.35–2.81). The present study identified few susceptible genotypes of the eNOS gene with the risk of hypertension. Moreover, the interactive effects between the environmental factors and the risk of hypertension were dependent on the eNOS genotypes.

## 1. Introduction

Nitric oxide (NO) produced from L-arginine by endothelial nitric oxide synthase (eNOS) plays a significant role in the regulation of vascular tone and in the control of blood pressure [1]. Reduction in basal NO release may predispose to hypertension, thrombosis, vasospasm, and atherosclerosis [2] and inhibition of eNOS elevates blood pressure in healthy humans [3]. Studies have shown that disruption of eNOS gene leads to hypertension in mice [4]. Furthermore, whole-body NO production in patients with essential hypertension is diminished under basal conditions [5]. Because of these important evidences of NO and eNOS involvement in the blood pressure regulation, the eNOS gene has therefore been studied as a putative candidate gene for hypertension. A number of eNOS gene polymorphisms have been identified so far. Among these, eNOS gene intron 4 ab polymorphism, eNOS gene exon 7 Glu298Asp variant (rs 1799983), and eNOS gene T786C polymorphism (rs 2070744) have been most studied for an association with hypertension. Although the role of eNOS gene polymorphisms in the incidence of hypertension seems to vary in different populations, only few studies on these polymorphisms have been conducted in Indian population [6, 7].

Moreover, the complex interplay between the environmental factors and genes for the risk of hypertension is still not clear and many dietary and lifestyle factors have been implicated in the aetiology of hypertension. In earlier studies, both tobacco use (either smoking/chewing or both) and alcohol consumption were associated with an increased risk of hypertension [8–10]. We, in our previous study [11], have illustrated a dose-response relation between the number of cigarettes smoked per day (*χ* ^2^ for trend = 26.07; *P* < 0.0001) and the amount of alcohol consumption per day (*χ* ^2^ for trend = 24.26; *P* < 0.0001) and the risk of hypertension. The same study also calculated the population attributable risk (PAR) and were found to be 70.3% (95% CI 63.0–77.5) for tobacco use, 45.3% (95% CI 37.1–53.4) for tobacco chewing, 31.5% (95% CI 21.3–40.9) for smoking, and 33.6% (95% CI 22.9–44.4) for alcohol consumption.

This prompted us to conduct an investigation to study the association between the polymorphisms of the endothelial nitric oxide synthase gene and hypertension in the tea garden worker community of northeastern India where the prevalence of hypertension is high [12].

## 2. Materials and Methods

### 2.1. Study Area and Study Subjects

The study area and the study population were identical with that described previously [11].

#### 2.1.1. Study Area

Dibrugarh, the largest tea exporting district in India and has the largest land area of tea cultivation among all the districts of Assam. In view of the importance of Dibrugarh district in the tea industry, the tea gardens in the Dibrugarh district were selected to conduct the present study.

#### 2.1.2. Study Subjects

The study is a case-control study and 700 subjects (350 cases and 350 age matched controls) from the tea garden worker community in the age group 20–65 years after obtaining signed informed consent were enrolled from ten randomly selected tea gardens of Dibrugarh district, Assam, India.

*Definition of Hypertension for the Present Study*. The hypertension status of the subjects was defined by the criteria formulated by the US Seventh Joint National Committee on Detection, Evaluation and Treatment of Hypertension (JNC-VII), that is, SBP ≥ 140 mmHg and/or DBP ≥ 90 mmHg and those on antihypertensive medication [13]. Initial identification of the cases was based on the hospital records maintained in the tea garden hospital and subsequently rechecked before enrolment. For this, three blood pressure readings by mercury sphygmomanometer were taken at an interval of ten minutes and the mean of the three readings was taken for categorizing the subjects. 

*Exclusion Criteria Adopted for the Present Study*. The exclusion criteria adopted during recruitment of the study subjects included subject's previous history of cardiovascular disease, women receiving oral contraceptives or hormone replacement therapy, pregnant women or lactating mothers, and inability to give informed consent or comply with the study protocol and subjects above or below the desired age. 

*Ethical Issue*. Study was approved by the Institutional Ethical Committee (IEC) of Regional Medical Research Centre, NE Region, Indian Council of Medical Research, Dibrugarh, India. Selected subjects were briefed about the study protocol and informed and signed consent was obtained from each of the selected study subjects. 

*Collection of Epidemiological Data*. A pretested peer-reviewed proforma containing questions pertaining to the demographic profile, socioeconomic status, and educational status was used to interview the study subjects. The anthropometric measurements included height and weight. Height was measured to the nearest centimeter using a stadiometer and weight to the nearest kilogram using a weighing machine. Body weight was measured in light clothing without shoes. Body mass index (BMI) was calculated using the formula weight in kg/height in meter squared and was categorized as underweight (<18 kg/m^2^), normal (18.0–24.9 kg/m^2^), overweight (25.0–29.9 kg/m^2^), or obese (≥30 kg/m^2^) according to the 2000 WHO criteria [14].

The study population had the habit of taking extra salt (as a side dish) which means the raw salt, taken as such, as a dish in addition to the salt that is used in the preparation of the meal by the side of their plate during lunch and dinner and eating it during the course of their meal either alone or with a chilli.

In the study population, tobacco is consumed mainly in two forms: smoked tobacco in the form of bidi, cigarette and smokeless tobacco chewing in the form of khaini (a tobacco quid containing a mixture of tobacco and lime with or without betel nut). A tobacco user was defined as one who has consumed tobacco in any form (cigarette, bidi, or chewed tobacco) in the past one month. A tobacco chewer was defined as one who consumed tobacco orally and included* khaini *alone or along with betel quidwith or without lime, pan and pan-masala or gutka (containing chewable tobacco). A smoker was defined as one who has consumed smoked tobacco in the form of bidi, cigarette, or any other forms of tobacco smoking in the past one month. A nonuser of tobacco was defined as one who has never consumed tobacco in any form (cigarette, bidi, or chewed tobacco). Those who did not consume tobacco in any form in the past one year were defined as past users of tobacco.

Local brew (locally known as* haria*) prepared from rice and other herbal ingredients was the most common alcohol drink. We categorized the habit of alcohol consumption into the following categories—nonuser, past user, occasional user, and moderate user. An alcohol user was defined as one who consumed alcohol in any form in the past one month. A nonuser of alcohol was defined as one who has never consumed alcohol in any form. Those who did not consume alcohol in any form in the past one year were defined as past users of alcohol. An occasional user of alcohol was defined as one who took 1–5 drinks a week whereas a moderate user of alcohol was defined as one who consumed ≥2 drinks daily. 

*Biochemical Investigations*. The biochemical indicators included fasting blood sugar (FBS) and renal function tests (blood urea and serum creatinine) and lipid profile tests. The 12-hour fasting blood samples for the glucose assay were collected in the morning into tubes containing NaF, and plasma glucose concentrations were determined using the glucose oxidase and peroxidase method [15]. Blood for the renal function tests was collected in sterile empty vials. Blood urea was estimated by enzymatic method [16] while serum creatinine was estimated by alkaline Jaffe's Picrate method [17] whereas total cholesterol, HDL cholesterol, and serum triglycerides were analyzed enzymatically with commercially available reagents. For participants, LDL cholesterol levels were calculated from the Friedewald equation (LDL cholesterol = total cholesterol − HDL cholesterol − triglycerides/5). All these laboratory parameters were analyzed by using commercially available Randox reagent kits and with help of a fully automated biochemical analyzer (Model-Fully, Biochemical System International, Italy). 

*Measurement of Blood Pressure*. The intent and purpose of the blood pressure measurement was explained to the study subjects in a reassuring manner and every effort was made to put the subject at ease. A standard mercury sphygmomanometer was used to measure the blood pressure with the subject in the sitting posture. The blood pressure was recorded in the right arm using a cuff of appropriate size with the instrument at the level of the subject's heart. The first and fifth Korotkoff sounds were taken as the systolic blood pressure and diastolic blood pressure, respectively. Three readings were taken at an interval of 10 minutes and the average of the three readings was taken to determine the hypertension status of the subject. The hypertension status of the subjects was defined by the criteria formulated by the US Seventh Joint National Committee on Detection, Evaluation and Treatment of Hypertension (JNC-VII), that is, SBP ≥ 140 mmHg and/or DBP ≥ 90 and those on antihypertensive medication [13].

### 2.2. Genetic Analysis

#### 2.2.1. eNOS Gene Intron 4 ab Polymorphism

The eNOS gene intron 4 ab polymorphism was determined bypolymerase chain reactionusing forward primer 5′ AGG CCC TAT GGT AGT GCC TTT 3′ and reverse primer 5′ TCT CTT AGT GCT GTG GTC AC 3′ to amplify the “a” and “b” alleles, which will result in the amplification of 393 bp and 420 bp products, respectively. Polymerase chain reaction (PCR) consisted of 0.5 mM of each primer (Sigma), 10 mM Tris-HCL pH 9.0 (Bangalore Genei), 0.2 mM dNTPs (Bangalore genei), 1 U of Taq DNA polymerase (Bangalore Genei), and 50–100 ng of genomic DNA following standardized protocol with slight modifications [18]. The PCR reactions are amplified using a Thermal Cycler (Gene Amp. PCR Systems 9700: Version 3.08, Applied Biosystems) at the following conditions: initial denaturation at 94.0°C for 5 minutes, 32 cycles of denaturation at 94.0°C for 1 minute, annealing temperature at 57.0°C for 1 minute, and extension at 72.0°C for 2 minutes followed by final extension at 72.0°C for 7 minutes. Amplification of PCR products is confirmed by electrophoresis on 3.5% agarose gel. The eNOS intron 4 a/b polymorphism results in three genotypes, namely, aa homozygous (aa), bb homozygous (bb), and ab heterozygous (ab).

#### 2.2.2. eNOS Gene Exon 7 Glu298Asp Variant (rs 1799983)

To genotype the G894T polymorphism in the exon 7, we performed a polymerase chain reaction (PCR) amplification of 206 bp fragment encompassing the variant, followed by restriction fragment length polymorphism (RFLP) using the restriction endonuclease* Mbo1* (Promega). Polymerase chain reaction consisted of 0.5 mM of each primer (Sigma), 10 mM Tris-HCL pH 9.0 (Bangalore Genei), 0.2 mM of dNTPs (Bangalore Genei), 2.4 U of Taq DNA polymerase (Bangalore Genei), and 50–100 ng of genomic DNA. The PCR primers were 5′ CAT GAG GCT CAG CCC CAG AAC 3′ (sense) and 5′ AGT CAA TCC CTT TGG TGC TCA C 3′ (antisense). The PCR amplification conditions consisted of 94°C for 5 minutes, followed by 40 cycles of 94°C for 30 seconds, 66°C for 30 seconds, 72°C for 30 seconds, and then 72°C for 8 minutes. The PCR amplicons were then incubated at 37°C for 3 hours. The 206 bp amplicon containing a thymine at nucleotide position 894 (corresponding to an aspartic acid at amino acid position 298) was cleaved into two fragments of 119 bp and 87 bp in length by* Mbo1 *digestion but not for a guanine in this position. The restriction digest products were analyzed by electrophoresis on a 2.5% agarose gel [19].

#### 2.2.3. eNOS Gene T-786C Polymorphism (rs 2070744)

The presence of the T → C conversion at nucleotide position 786 in the 5′-flanking region of the eNOS gene was determined by PCR amplification with the primers 5′-ATG CTC CCA CCA GGG CAT CA-3′ (sense) and 5′-GTC CTT GAG TCT GAC ATT AGG G-3′ (antisense) followed by restriction fragment length polymorphism (RFLP) using the restriction endonuclease* Ng0AIV *(Promega). Polymerase chain reaction consisted of 0.5 mM of each primer (Sigma), 10 mM Tris-HCL pH 9.0 (Bangalore Genei), 0.2 mM of dNTPs (Bangalore Genei), 2.4 U of Taq DNA polymerase (Bangalore Genei), and 50–100 ng of genomic DNA. The PCR amplification conditions consisted of 94°C for 6 minutes, followed by 40 cycles of 94°C for 30 seconds, 65°C for 30 seconds, 72°C for 1 minute, and then 72°C for 8 minutes. The 236 bp PCR fragments were digested with* Ng0AIV* restriction enzyme for 4 hours at 37°C. The T-allele has no* Ng0AIV *cleavage site, whereas the PCR product is cleaved into two fragments of 203 bp and 33 bp in the presence of the C^786^ allele [20].

### 2.3. Statistical Analysis

The software Statistical Package for Social Sciences (SPSS) version 13.0 and Epi-Info 2002 (CDC, Atlanta, USA) are used in the analysis of the data generated. A *P* value (two-sided) ≤ 0.05 was considered as statistically significant. Results are presented as number and percentage or mean ± SD. Chi-square test was used to test the association between categorized variables and independent *t*-test and ANOVA was used to compare means of continuous variables. The crude (unadjusted) relationship between the exposure variables and the risk of hypertension was examined in univariate logistic regression analyses. Multivariate logistic regression analysis was done to evaluate the simultaneous effects of various exposure variables, with adjustment for the potential confounding effects of other factors. The frequencies for all the possible haplotypes were calculated with the cubic exact solutions for the estimation of pairwise haplotype frequencies (http://www.oege.org/software/cubex) [21]. This programme estimates the haplotype frequencies, Lewontin's standardized disequilibrium coefficient (*D*′), and squared correlation coefficient (*r* ^2^) for pairwise linkage disequilibrium between two loci.

## 3. Results

The study was designed as an age-adjusted case-control study and included a total of 700 study participants (350 cases and 350 controls) in the age group 20–65 years of both sexes. Distribution of sociodemographic and clinical characteristics of the study population has been shown in Table 1.

Genotype and allelic frequencies for the eNOS gene polymorphisms for eNOS gene intron 4 ab polymorphism and eNOS gene exon 7 Glu298Asp variant were in agreement with the frequencies predicted by the Hardy-Weinberg equilibrium (*χ* ^2^ = 1.12, df = 2, *P* > 0.05 and *χ* ^2^ = 2.67, df = 2, *P* > 0.05, resp.).

To test the association of the eNOS gene polymorphisms with hypertension, genotypic odds ratios (ORs) were calculated (Table 2). In univariate logistic regression analysis, eNOS intron 4 aa genotype was found to be associated with increased risk of hypertension. In multivariate model of logistic regression too, the risk persisted even after adjustment for age, sex, extra salt intake, smoking, tobacco chewing, and habit of alcohol consumption (eNOS intron 4 aa genotype (OR 6.81; 95% CI 2.29–20.25) and eNOS intron 4 ab genotype (OR 2.23; 95% CI 1.55−3.20)).

In univariate logistic regression analysis, eNOS 894TT genotype was found to be a risk factor. In multivariate model of logistic regression, adjusted for age, sex, extra salt intake, smoking, tobacco chewing, and habit of alcohol consumption, the eNOS 894TT genotype (OR 7.84; 95% CI 2.57–23.96) and eNOS 894GT genotype (OR 3.98; 95% CI 2.65–5.98) were at an increased risk of hypertension.

Pairwise comparison of the three studied polymorphisms (eNOS intron 4ab, eNOS exon 7, and eNOS T-786C) of eNOS genedepicting LD measures is presented in Table 3. We observed linkage disequilibrium between eNOS intron 4 and eNOS exon 7 pairs (*D*′ = −0.144; *r* ^2^ = 0.172; *χ* ^2^ = 12.04; *P* < 0.0001).

The associations of the possible haplotypes of the three polymorphisms of the eNOS gene with the risk of hypertension in the study population are presented in Table 4. The haplotypes aT, aG, and bT of the variants eNOS-4 and eNOS-7, aT of the variants eNOS intron 4 and eNOS T-786C, and TT and TC of the variants eNOS exon 7 and eNOS T-786C were found to be significantly associated with the risk of hypertension in the study population.

We performed stratified analysis to study interaction or effect modification according to eNOS gene polymorphism.

The risk of hypertension was increased among tobacco users (either smoking/chewing or both) carrying eNOS intron 4 aa genotype (OR 14.00: 95% CI 1.20−163.37) in comparison with the subjects who had the habit of tobacco use (either smoking/chewing or both) belonging to eNOS intron 4 ab genotype (OR 8.06: 95% CI 4.41−14.73) and eNOS intron 4 bb genotype (OR 4.09: 95% CI 2.74−6.12). Similarly, the risk of hypertension was higher among subjects who had the habit of alcohol consumption carrying eNOS intron 4 aa genotype (OR 12.00: 95% CI 1.20–143.73) in comparison with the subjects who had the habit of alcohol consumption carrying eNOS intron 4 ab genotype (OR 1.56: 95% CI 0.91−2.65) and eNOS intron 4 bb genotype (OR 2.07: 95% CI 1.41−3.04) (Table 5).

Risk of hypertension was increased among subjects who had the habit of tobacco use (either smoking/chewing or both) carrying eNOS 894GG genotype (OR 5.56: 95% CI 3.72−8.31) in comparison with the subjects who had the habit of tobacco use (either smoking/chewing or both) with eNOS 894GT genotype (OR 3.86: 95% CI 1.95−7.67) and eNOS 894TT genotype (OR 2.29: 95% CI 0.27–19.66). Similarly, the risk of hypertension was increased among subjects with eNOS 894GG genotype who consume alcohol (OR 1.95: 95% CI 1.35−2.81) in comparison to those who consume alcohol with eNOS 894GT genotype (OR 2.41: 95% CI 1.24−4.69) and eNOS 894TTgenotype (OR 1.09: 95% CI 0.13−9.12) (Table 5).

The risk of hypertension was increased among subjects who had the habit of tobacco use (either smoking/chewing or both) carrying eNOS T-786C CC genotype (OR 9.00: 95% CI 1.14–71.04) in comparison with the subjects who had the habit of tobacco use (either smoking/chewing or both) carrying eNOS T-786C TC genotype (OR 4.51: 95% CI 2.69–7.56) and eNOS T-786C TT genotype (OR 5.84: 95% CI 3.79−8.98) (Table 5).

## 4. Discussion

We conducted an age-adjusted case-control study to explore the association between the endothelial nitric oxide synthase gene polymorphisms and hypertension in the tea garden population of northeastern India in the age group 20–65 years of both sexes.

In the present study the frequency of the a-allele and b-allele was found to be 0.20 and 0.80, respectively. The “b”-allele frequency observed in our study is similar to studies conducted in Japanese and UK populations [22, 23].

The eNOS intron 4 aa genotype in the present study has been associated with an increased risk of hypertension in comparison with the eNOS intron 4 ab genotype and eNOS intron 4 bb genotype. Although the eNOS intron 4 ab polymorphism is an intronic polymorphism, it is reported that the eNOS intron 4 ab polymorphism modulate transcription, influencing translation efficiency, mRNA stability, and enzyme levels [24]. Further a meta-analysis of 35 genetic studies, also, supported the association between eNOS intron 4 ab polymorphism and hypertension [25]. These findings highlight the significance of the eNOS intron 4 ab polymorphism in the development of hypertension.

As reported from different parts of India, (north Indian [6] and south Indian populations [26]),the eNOS exon 7 homozygous GG genotype (eNOS 894GG) (70.0%) was predominant followed by eNOS exon 7 heterozygous GT genotype (eNOS 894GT) (26.14%) and eNOS exon 7 homozygous TT genotype (eNOS 894TT) (3.86%).

A low (3.86%) frequency of the homozygous mutant eNOS 894TT genotype was observed. The study revealed a “T” allele frequency of 0.17 among the tea garden community which is comparable to that observed among south Indian (0.13) [26] and north Indian populations (0.15) [6].

Present study demonstrated a significant association between eNOS exon 7 894TT genotype and the risk of hypertension. These results indicate that eNOS gene exon 7 Glu298Asp variant plays an important role in blood pressure regulation and may be a risk factor of hypertension for the tea garden community of Assam. The production of basal nitric oxide is significantly decreased in hypertensive cases as compared to healthy controls [27]. The eNOS gene exon 7 Glu298Asp variant causes a Glu298 change to 298Asp which alters the structure of eNOS and affects its activity by decreasing the production of nitric oxide and ultimately increasing blood pressure [28]. Similarly a significantly higher frequency of the T allele has been found to be associated with hypertension [29] and higher blood pressure levels [30] in Japanese subjects.

In the study, no association was detected between eNOS gene T-786C polymorphism and hypertension as also reported elsewhere [31–33].

Individual gene polymorphisms may not be consistent and reliable risk markers for developing hypertension and haplotypes can sometimes provide greater power than single-marker analyses for genetic disease associations. Tanus-Santos and his team investigated the role eNOS haplotypes in susceptibility to cardiovascular diseases and reported marked interethnic differences in the distribution of eNOS gene polymorphisms, haplotype frequency, and the association between the eNOS variants in Caucasians and African-Americans and in white and black Brazilians [34, 35]. The present study considered the pairwise comparison of the three polymorphisms of the eNOS gene (namely, eNOS intron 4 ab, eNOS exon 7, and eNOS T-786C) observed a significant linkage disequilibrium between eNOS intron 4 and eNOS exon 7 pairs.

The haplotypes aT, aG, and bT of the variants eNOS-4 and eNOS-7, aT of the variants eNOS intron 4 and eNOS T-786C, and TT and TC of the variants eNOS exon 7 and eNOS T-786C were found to be significantly associated with the risk of hypertension in the study population. Our study does not reveal the mechanism for the association of hypertension with specific eNOS haplotypes but is supported by other studies that reported a major influence of the eNOS haplotypes on disease risks [36–39]. The study conducted by Sandrim and his group (2006) [40] reported a protective effect for the “C-Glu-b” haplotype against hypertension and that the “C-Asp-b” haplotype increases the susceptibility to hypertension. Moreover, their results suggested that eNOS haplotypes were not associated with resistance to antihypertensive therapy. Another study [41] from the same group suggested a protective role for eNOShaplotype “C-Glu-b” against the development of hypertension and that the haplotype “C-Asp-b” increases the susceptibility to hypertension in patients with or without type 2 diabetes mellitus. Our findings suggest a contribution of eNOS haplotypes to the development of hypertension that may be obscured when specific eNOS genotypes alone are considered.

The complex interplay between genes and environmental factors affecting blood pressure regulation is not well understood. It is the coexistence of adverse environmental factors on the background of genetic susceptibility that determines the initiation and progression of hypertension. Because these exposures are modifiable, their interaction with genetic susceptibility to hypertension is of substantial public health importance.

The study also documented a significant gene-environment interaction between eNOS intron 4 aa genotype and the habit of alcohol consumption for the risk of hypertension in the study population. Mechanisms underlying the relationship between alcohol and blood pressure remain ambiguous, though several mechanisms have been proposed [42, 43]. Some suggested mechanisms include stimulation of the sympathetic nervous system, inhibition of nitric oxide, depletion of ions, and increased intracellular calcium especially in vascular smooth muscle.

An interesting surprising finding of the present study was a significant gene-environment interaction between eNOS exon 7 894GG genotype and behavioral risk factors like tobacco chewing and alcohol consumption for the risk of hypertension. The molecular effect of the eNOS exon 7 Glu298Asp polymorphism on eNOS enzyme function is still not clear. Lacolley et al. [44] reported 894G allele to be associated with an increased risk of hypertension in Caucasians. In the present study we observed a significant linkage disequilibrium between eNOS intron 4 and eNOS 7 pair and found the haplotype aG of the variants eNOS-4 and eNOS-7 to be significantly associated with the risk of hypertension. This may be a possible explanation. However, further studies to assess to gene-environment relationship between eNOS 894GG genotype and the habit of alcohol consumption in the pathogenesis of hypertension are warranted.

Some of the limitations faced during the study should, also, be considered. During assessment of demographic variables, we adopted a recall method that may introduce some bias in estimations of demographic variables. A small proportion (<2.0%) dropout due to nonavailability of consent for blood sample collection occurred in the study.

## 5. Conclusion

The present study detected the association of endothelial nitric oxide synthase gene polymorphisms with hypertension and identified few susceptible genotypes of the endothelial nitric oxide synthase gene with the risk of hypertension. The present results suggested that eNOS gene variants and their interactions with some environmental risk factors play an important role in the pathophysiology of hypertension. The understanding of such mechanisms may help better appreciate the molecular basis of hypertension.

## Figures and Tables

**Table 1:** Distribution of sociodemographic and clinical characteristics of the study population.

Variable	Study subjects (*N* = 700)		*P* value
	Control (*n* = 350)	Case (*n* = 350)	
Age (mean ± SD)	36.2 ± 12.3	36.4 ± 12.3	0.806
Systolic blood pressure (mmHg; mean ± SD)	116.3 ± 9.8	153.6 ± 19.6	0.000∗
Diastolic blood pressure (mmHg; mean ± SD)	74.7 ± 6.8	92.9 ± 9.9	0.000∗
Sex			
Male	143 (40.9)	134 (38.3)	0.536
Female	207 (59.1)	216 (61.7)	
BMI			
Underweight	117 (33.4)	103 (29.4)	0.005∗
Normal	228 (65.1)	224 (64.0)	
Overweight	4 (1.1)	15 (4.3)	
Obese	1 (0.3)	8 (2.3)	
Alcohol intake			
Yes	155 (44.3)	213 (60.9)	0.000∗
Alcohol consumption			
Nonuser	195 (55.7)	137 (39.1)	0.000∗
Past user	26 (7.4)	18 (5.1)	
1–5 drinks per week	75 (21.4)	103 (29.4)	
≥2 drinks daily	54 (15.4)	92 (26.3)	
Habit of tobacco use			
Tobacco user	126 (36.0)	262 (74.9)	0.000∗
Smoking habit			
Yes	58 (16.6)	112 (32.0)	0.000∗
Frequency of smoking			
Nonuser	292 (83.4)	238 (68.0)	0.000∗
Past user	11 (3.1)	12 (3.4)	
Rare	11 (3.1)	20 (5.7)	
1–4 nos/day	30 (8.6)	56 (16.0)	
5–10 nos/day	4 (1.1)	14 (4.0)	
More than 10 nos/day	2 (0.6)	10 (2.9)	
Tobacco chewer			
Yes	91 (26.0)	183 (52.3)	0.000∗
Blood glucose (mg/dL; mean ± SD)	96.4 ± 17.5	104.9 ± 24.2	0.000∗
Blood urea (mg/dL; mean ± SD)	21.3 ± 6.5	23.7 ± 8.3	0.000∗
Serum creatinine (mg/dL; mean ± SD)	0.9 ± 0.5	0.9 ± 0.3	0.000∗
Serum sodium (mmol/L; mean ± SD)	140.7 ± 7.5	146.2 ± 8.5	0.000∗
Serum potassium (mmol/L; mean ± SD)	6.5 ± 2.1	5.5 ± 1.5	0.000∗
Serum cholesterol (mg/dL; mean ± SD)	144.8 ± 16.2	146.3 ± 38.9	0.003∗
Serum HDL cholesterol (mg/dL; mean ± SD)	41.9 ± 7.4	40.38 ± 6.62	0.000∗
Serum triglycerides (mg/dL; mean ± SD)	140.8 ± 38.9	148.4 ± 36.7	0.027∗
Serum LDL (mg/dL; mean ± SD)	68.6 ± 16.6	73.9 ± 22.5	0.059

All values within parenthesis are percentages.

SD: standard deviation.

BMI: body mass index.

HDL: high density lipoprotein.

LDL: low density lipoprotein.

∗Statistically significant (*P* value ≤ 0.05).

**Table 2:** Endothelial nitric oxide synthase (eNOS) gene polymorphisms and the risk of hypertension in the study population.

eNOS gene polymorphisms		Control (*n* = 350)	Case (*n* = 350)	Crude OR (95% CI)	*P* value	Adjusted OR^#^(95% CI)	*P* value
eNOS gene intron 4 ab polymorphism	bb	256 (73.2%)	190 (54.3%)	1.000	—	1.000	—
	ab	89 (25.4%)	142 (40.6%)	2.15 (1.55–2.97)	0.002∗	2.23 (1.55–3.20)	0.000∗
	aa	5 (1.4%)	18 (5.1%)	4.85 (1.77–13.30)	0.000∗	6.81 (2.29–20.25)	0.001∗
	b	601 (85.9%)	522 (74.6%)	1.000	—	—	—
	a	99 (14.1%)	178 (25.4%)	2.07 (1.56–2.74)	0.000∗	—	—
		(aa + ab)/bb		2.29 (1.67–3.15)	0.000∗	2.33 (1.65–3.30)	0.000∗
		aa/(ab + bb)		3.74 (1.37–10.19)	0.010∗	3.85 (1.34–11.03)	0.012∗
	Homozygous for rare allele versus homozygous for common allele			4.85 (1.77–13.30)	0.002∗	6.81 (2.29–20.25)	0.001∗
	Heterozygous for the common allele versus homozygous for the common allele			2.15 (1.55–2.97)	0.000∗	2.21 (1.55–3.15)	0.000∗

eNOS gene exon 7 Glu298Asp polymorphism	GG	296 (84.6%)	194 (55.4%)	1.000	—	1.000	—
	GT	50 (14.3%)	133 (38.0%)	4.06 (2.80–5.89)	0.000∗	3.98 (2.65–5.98)	0.000∗
	TT	4 (1.1%)	23 (6.6%)	8.77 (2.99–25.76)	0.000∗	7.84 (2.57–23.96)	0.000∗
	G	642 (91.7%)	521 (74.4%)	1.000	—	—	—
	T	58 (8.3%)	179 (25.6%)	3.80 (2.74–5.29)	0.000∗	—	—
		(TT + GT)/GG		4.41 (3.08–6.31)	0.000∗	4.25 (2.90–6.22)	0.000∗
		TT/(GT + GG)		6.08 (2.08–17.78)	0.001∗	6.35 (2.10–19.22)	0.001∗
	Homozygous for rare allele versus homozygous for common allele			8.77 (2.99–25.76)	0.000∗	7.84 (2.57–23.96)	0.000∗
	Heterozygous for the common allele versus homozygous for the common allele			4.06 (2.80–5.89)	0.000∗	3.88 (2.60–5.78)	0.000∗

eNOS gene T786C polymorphism	TT	214 (61.1%)	200 (57.1%)	1.000	—	1.000	—
	TC	127 (36.3%)	139 (39.8%)	1.17 (0.86–1.59)	0.315	1.19 (0.85–1.66)	0.324
	CC	9 (2.6%)	11 (3.1%)	1.31 (0.53–3.22)	0.560	1.41 (0.52–3.81)	0.497
	T	555 (79.3%)	539 (77.0%)	1.000	—	—	—
	C	145 (20.7%)	161 (23.0%)	1.14 (0.88–1.49)	0.301	—	—
		(CC + TC)/TT		1.18 (0.87–1.60)	0.282	1.21 (0.87–1.67)	0.255
		CC + (TC + TT)		1.23 (0.50–3.01)	0.651	1.25 (0.48–3.24)	0.647
	Homozygous for rare allele versus homozygous for common allele			1.31 (0.53–3.22)	0.560	1.41 (0.52–3.81)	0.497
	Heterozygous for the common allele versus homozygous for the common allele			1.17 (0.86–1.59)	0.315	1.19 (0.85–1.66)	0.324

OR: odds ratio.

CI: confidence interval.

^#^Adjusted for age, sex, extra salt intake, smoking, tobacco chewing and habit of alcohol consumption, and eNOS gene polymorphisms.

∗Statistically significant (*P* value ≤ 0.05).

**Table 3:** Measures of linkage disequilibrium observed in a pairwise comparison of the two polymorphisms of the endothelial nitric oxide synthase (eNOS) gene among the study population.

Variant 1	Variant 2	*D*′	*r* ^2^	*χ* ^2^	Linkage disequilibrium
eNOS 4	eNOS 7	−0.144	0.0172	12.04	In linkage disequilibrium
eNOS 4	eNOS T786C	0.125	0.0043	3.01	Not in linkage disequilibrium
eNOS 7	eNOS T786C	−0.309	0.0054	3.78	Not in linkage disequilibrium

*D*′: Lewontin's standardized disequilibrium coefficient.

*r* ^2^: squared correlation coefficient for pairwise linkage disequilibrium between two loci.

*χ* ^2^: Chi-square value.

**Table 4:** Haplotype frequency distribution (case versus control) of the three polymorphisms of the endothelial nitric oxide synthase (eNOS) gene in the study population.

Variants	Haplotype	Haplotype frequency		OR (95% CI)	*P* value
		Case (*n* = 350)	Control (*n* = 350)		
eNOS 4 and eNOS 7	**bG**	0.5550	0.7912	0.33 (0.23–0.46)	0.000∗
	**aT**	0.0650	0.0112	6.08 (1.97–20.99)	0.002∗
	**aG**	0.1850	0.1288	1.55 (1.00–2.39)	0.048∗
	**bT**	0.1950	0.0688	3.28 (1.95–5.52)	0.000∗

eNOS 4 and eNOS T786C	**bT**	0.5775	0.6794	0.64 (0.47–0.89)	0.006∗
	**bC**	0.1725	0.1806	0.94 (0.63–1.42)	0.843
	**aT**	0.1925	0.1106	1.89 (1.21–2.96)	0.004∗
	**aC**	0.0575	0.0294	2.17 (0.96–5.02)	0.07

eNOS 7 and eNOS T786C	**GT**	0.5698	0.7268	0.50 (0.36–0.69)	0.000∗
	**GC**	0.1702	0.1932	0.86 (0.57–1.28)	0.47
	**TT**	0.2002	0.0632	3.73 (2.19–6.38)	0.000∗
	**TC**	0.0598	0.0168	3.66 (1.38–10.26)	0.006∗

OR: odds ratio.

CI: confidence interval.

∗Statistically significant (*P* value ≤ 0.05).

**Table 5:** Stratified analysis to study the relation between endothelial nitric oxide synthase (eNOS) gene polymorphisms and the risk of hypertension in the subgroups with selected habits in the study population.

eNOS gene polymorphism	Parameter		Odds ratio (95% CI)	*P* value
eNOS gene intron 4 ab polymorphism	Tobacco use (either smoking/chewing or both)	Subjects who have the habit of tobacco use (either smoking/chewing or both) with eNOS4 aa genotype	14.00 (1.20–163.37)	0.035∗
		Subjects who have the habit of tobacco use (either smoking/chewing or both) with eNOS4 ab genotype	8.06 (4.41–14.73)	0.000∗
		Subjects who have the habit of tobacco use (either smoking/chewing or both) with eNOS4 bb genotype	4.09 (2.73–6.12)	0.000∗
	Alcohol consumption	Subjects who have the habit of alcohol consumption with eNOS4 aa genotype	12.00 (1.20–143.73)	0.035∗
		Subjects who have the habit of alcohol consumption with eNOS4 ab genotype	1.56 (0.91–2.65)	0.105
		Subjects who have the habit of alcohol consumption with eNOS4 bb genotype	2.07 (1.41–3.04)	0.000∗

eNOS gene exon 7 Glu298Asp polymorphism	Tobacco use (either smoking/chewing or both)	Subjects who have the habit of tobacco use (either smoking/chewing or both) with eNOS7 GG genotype	5.56 (3.72–8.31)	0.000∗
		Subjects who have the habit of tobacco use (either smoking/chewing or both) with eNOS7 GT genotype	3.86 (1.95–7.67)	0.000∗
		Subjects who have the habit of tobacco use (either smoking/chewing or both) with eNOS7 TT genotype	2.29 (0.27–19.66)	0.451
	Alcohol consumption	Subjects who have the habit of alcohol consumption with eNOS7 GG genotype	1.95 (1.35–2.81)	0.000∗
		Subjects who have the habit of alcohol consumption with eNOS7 GT genotype	2.41 (1.24–4.69)	0.009∗
		Subjects who have the habit of alcohol consumption with eNOS7 TT genotype	1.09 (0.13–9.12)	0.936

eNOS gene T786C polymorphism	Tobacco use (either smoking/chewing or both)	Subjects who have the habit of tobacco use (either smoking/chewing or both) with eNOS T786C TT genotype	5.84 (3.79–8.98)	0.000∗
		Subjects who have the habit of tobacco use (either smoking/chewing or both) with eNOS T786C TC genotype	4.51 (2.69–7.56)	0.000∗
		Subjects who have the habit of tobacco use (either smoking/chewing or both) with eNOS T786C CC genotype	9.00 (1.14–71.04)	0.037∗
	Alcohol consumption	Subjects who have the habit of alcohol consumption eNOS T786C TT genotype	2.07 (0.47–3.06)	0.547
		Subjects who have the habit of alcohol consumption with eNOS T786C TC genotype	1.75 (0.18–2.86)	0.084
		Subjects who have the habit of alcohol consumption with eNOS T786C CC genotype	2.92 (0.41–20.90)	0.287

∗Statistically significant (*P* value ≤ 0.05).

CI: confidence interval.
